# Schistosome-derived microRNAs: promise, pitfalls and priorities for molecular diagnosis

**DOI:** 10.1186/s40249-026-01425-w

**Published:** 2026-02-25

**Authors:** Haoran Zhong, Guiquan Guan, Yamei Jin

**Affiliations:** 1 National Reference Laboratory for Animal Schistosomiasis, Key Laboratory of Animal Parasitology of Ministry of Agriculture and Rural Affairs, Shanghai Veterinary Research Institute, Chinese Academy of Agricultural Sciences, Shanghai, People’s Republic of China; 2 State Key Laboratory for Animal Disease Control and Prevention, Key Laboratory of Veterinary Parasitology of Gansu Province, Lanzhou Veterinary Research Institute, Chinese Academy of Agricultural Science, Lanzhou, Gansu People’s Republic of China

**Keywords:** Schistosomiasis, microRNA, Extracellular vesicles, Diagnosis, Policy integration

## Abstract

**Background:**

Schistosomiasis remains a major neglected tropical disease with persistent transmission despite decades of control programs. Recent discoveries of schistosome-derived microRNAs (miRNAs) have introduced new opportunities for precise, non-invasive molecular diagnosis. These miRNAs, often encapsulated in extracellular vesicles (EVs) or associated with Argonaute proteins, are stable in host biofluids and reflect active infection, worm burden, and pathological status. Therefore, this commentary aims to summarize current advances in schistosome-derived miRNAs for molecular diagnosis, discuss the major challenges limiting their field deployment, and propose future priorities for translational development.

**Main text:**

Accumulating evidence from both experimental models and human studies supports the diagnostic value of schistosome-derived miRNAs, with several candidates demonstrating high sensitivity and specificity across *Schistosoma* species. However, despite this promise, translation to field applications remains limited. Major challenges include low detection sensitivity in low-intensity infections, lack of standardized reference materials, absence of harmonized workflows, and dependence on complex laboratory infrastructure. Structural barriers—such as insufficient policy support, weak diagnostic infrastructure, and global inequities in resource allocation—further constrain deployment in endemic regions. Bridging these gaps demands a shift from exploratory discovery to translational and equity-centered development.

**Conclusions:**

In summary, although schistosome-derived microRNAs have demonstrated strong diagnostic potential across experimental and clinical settings, their impact remains limited by technological, infrastructural, and policy-related challenges, particularly in endemic regions. Future priorities should emphasize affordable, point-of-care diagnostic platforms, establishment of shared databases and international standards, and integration of molecular tools into national surveillance and elimination programs. Aligning technological innovation with accessibility and health equity will be crucial to transform schistosome miRNA biomarkers from laboratory findings into practical tools for global schistosomiasis control and elimination.

**Graphical Abstract:**

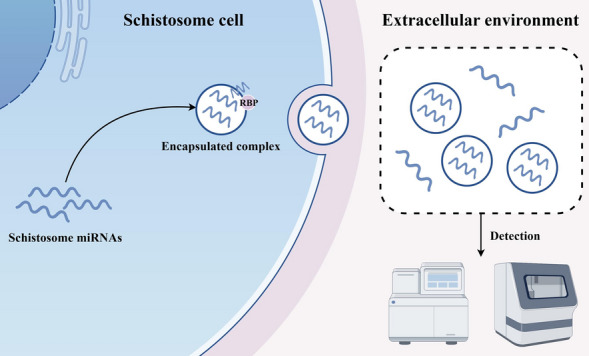

## Background

Schistosomiasis is a paradigmatic disease of poverty, disproportionately affecting populations in rural, water-associated, and resource-limited regions where access to safe water, sanitation, and healthcare is minimal [1]. It remains one of the most prevalent neglected tropical diseases recognized by the World Health Organization (WHO), with an estimated 250 million people infected and nearly one billion at risk across 78 endemic countries—predominantly in sub-Saharan Africa, Southeast Asia, and parts of Latin America [2]. The disease is transmitted through skin contact with freshwater harboring cercariae released from infected snails, linking its persistence directly to environmental, social, and economic determinants. Beyond its biological burden, schistosomiasis reflects profound inequities in global health—its distribution maps almost perfectly onto the contours of poverty [3].

Clinically, infection with *Schistosoma mansoni*, *S. haematobium*, or *S. japonicum* leads to chronic intestinal or urogenital disease and progressive organ-specific pathologies such as hepatic fibrosis, portal hypertension, and bladder carcinoma [4]. Despite decades of control efforts, including mass drug administration with praziquantel, reinfection rates remain high, especially where ecological and socioeconomic conditions continue to favor transmission. The WHO aims to eliminate schistosomiasis as a public health problem by 2030. Strengthening diagnostic capacity is therefore essential to support surveillance and guide interventions toward this target [2].

Traditional parasitological diagnosis, notably the Kato–Katz stool examination or urine filtration, continues to serve as the global field standard because of its simplicity and low cost. However, these methods have low sensitivity in cases of low-intensity or chronic infections and show high variability across samples and days. Antigen detection assays, such as circulating cathodic antigen (CCA) and circulating anodic antigen (CAA) tests, have improved sensitivity, particularly for *S. mansoni*, but their performance remains inconsistent due to antigen fluctuation, host immune variability, and the lack of reliable point-of-care formats for multiple *Schistosoma* species [5]. As countries advance from morbidity control to transmission interruption, more sensitive and quantitative diagnostic tools are required to identify residual infections and support surveillance.

Recent advances in RNA biology suggest that schistosome-derived microRNAs (miRNAs) may help address these challenges [6]. These small non-coding RNAs circulate stably in host biofluids, protected within extracellular vesicles (EVs) or associated with Argonaute proteins, and display species- and stage-specific expression. Such features make them attractive candidates for early, non-invasive, and quantitative diagnosis [7]. However, enthusiasm should be tempered by realism: while laboratory studies consistently highlight the diagnostic promise of schistosome-derived miRNAs, their translation into routine public health practice remains elusive. This article therefore offers a current opinion on the potential, limitations, and priorities of schistosome-derived miRNAs for diagnosis, arguing that the central question is no longer whether these molecules can serve as biomarkers, but how they can be made accessible, standardized, and equitably implemented in the field.

## Generation and secretion of schistosome-derived miRNAs

Similar to higher eukaryotes, the *Schistosoma* genome encodes a diverse repertoire of small non-coding RNAs, among which microRNAs (miRNAs) are predominant and play key regulatory roles [8, 9]. After maturation, schistosome miRNAs can be selectively packaged into EVs or secretory granules for release into the host environment, suggesting that their export is a tightly regulated process linked to parasite–host communication. While some miRNAs are involved in developmental and metabolic regulation, their secretion mechanisms and stabilization in extracellular spaces are crucial for understanding their biological functions and diagnostic relevance [10–14]. Recent work demonstrates that schistosome EV–derived miRNAs profoundly influence host immunity and fibrosis. These vesicles not only release small RNAs that weaken host anti-parasitic defenses—such as sja-bantam, miR-125b, and sma-miR-10, which promote macrophage proliferation or dampen Th2 cytokine signaling [15, 16]—but also secrete miRNAs that regulate the host’s fibrotic response, including pro-fibrotic miRNAs (e.g., sja-miR-1, sja-miR-2162, miR-30, and miR-33) that activate hepatic stellate cells through the Wnt/β-catenin and TGF-β pathways [17–20], and anti-fibrotic miRNAs (e.g., sja-miR-71a and sja-let-7) that limit inflammation and hepatic stellate cell activation [21, 22]. Together, these secretion mechanisms and the remarkable stability of EV-encapsulated miRNAs in host biofluids highlight their potential as circulating biomarkers for schistosomiasis diagnosis.

## Diagnostic potential of schistosome-derived miRNAs

Schistosome-derived miRNAs offer unique diagnostic advantages, including remarkable stability in circulation, species specificity, and the ability to reflect dynamic infection and treatment status [23, 24]. Nonetheless, their diagnostic application faces inherent challenges such as low abundance in biological fluids, short sequence length that limits assay design, and potential cross-reactivity with homologous host miRNAs [9]. Understanding and addressing these miRNA-specific challenges are crucial for realizing their full diagnostic potential.

Encapsulation within EVs further enhances the stability and detectability of circulating miRNAs [7]. Increasing research interest has been directed toward their diagnostic potential, with studies encompassing animal models, human cohorts, and multiple *Schistosoma* species. For instance, five *S. japonicum*-specific miRNA signatures—sja-bantam, sja-miR-3479, sja-miR-10, sja-miR-3096, and sja-miR-8185—were first identified in rabbit plasma through high-throughput small RNA sequencing and subsequently validated by qRT-PCR in infected mice [25]. Human investigations further demonstrated translational relevance, as sja-miR-2b-5p and sja-miR-2c-5p were detected in infected cohorts using qRT-PCR, exhibiting moderate diagnostic accuracy [26]. For *S. mansoni*, key miRNAs including sma-miR-277, sma-miR-3479-3p, and sma-bantam were quantified by qRT-PCR and showed diagnostic sensitivities and specificities approaching 80–90% in endemic populations [27]. In travelers infected with *S. mansoni*, *S. haematobium*, or *S. mekongi*, sma-bantam effectively distinguished cases from controls with good diagnostic performance (AUC ≈ 0.79) [28].

Beyond single miRNA markers, combinations of EV-derived miRNAs such as bantam, miR-2c-3p, and miR-3488—identified and validated using qRT-PCR—achieved AUC values exceeding 0.9, underscoring their strong diagnostic potential [28]. Collectively, these studies demonstrate that parasite-derived miRNAs, particularly those encapsulated within EVs, represent promising candidates for early diagnosis, post-treatment monitoring, and species differentiation.

Compared with other molecular diagnostic targets (e.g., DNA or protein antigens), miRNAs present a distinct set of opportunities and challenges. DNA-based assays, such as quantitative reverse transcription PCR (qRT-PCR) or loop-mediated isothermal amplification (LAMP), directly detect parasite genetic material and thus achieve excellent analytical sensitivity, but they require intact nucleic acids and high-quality extraction procedures that may not be feasible in all settings [29]. CCA and CAA tests detect schistosome-derived glycan antigens that reflect active infection status. These assays are well suited for field applications because they are rapid, minimally invasive, and can be performed using urine or serum samples without requiring complex laboratory infrastructure. However, their diagnostic performance may vary depending on host immune status and parasite species [30].

Reported sensitivities and specificities range from approximately 55% to over 90%, reflecting differences in infection intensity, host background, sample type, and detection methodology. These findings highlight both the promise and current limitations of miRNA-based diagnosis and underscore the need for standardized protocols, reference materials, and multicenter validation before clinical implementation.

## From bench to field: why promise has not met practice

Although miRNA-based molecular diagnostics offer unprecedented opportunities for schistosomiasis control, translating these advances into practical public health tools in endemic and resource-limited regions remains fraught with challenges. These obstacles stem not only from laboratory and infrastructural constraints but also from deeper structural tensions between scientific innovation and social systems.

Current detection of schistosome miRNAs, as reported in existing studies, relies primarily on RT-qPCR and next-generation sequencing (NGS). Among these, RT-qPCR is the most widely used technique owing to its high analytical sensitivity and throughput in quantifying known miRNAs. However, it requires precise normalization, a stable power supply, and skilled personnel, and its performance may be affected by inhibitors present in biological matrices. In contrast, NGS has been employed in several recent studies to enable comprehensive profiling and discovery of novel miRNA biomarkers, but its application remains limited by high cost, data intensity, and the need for advanced bioinformatics infrastructure—conditions rarely met in endemic settings [31].

Emerging isothermal amplification techniques such as LAMP and recombinase polymerase amplification (RPA) have also been explored for miRNA detection, yet their application remains technically challenging because of the short and structured nature of miRNAs [32]. Modified strategies employing stem–loop primers, hybridization chain reaction, or padlock probe–based amplification may enable adaptation of these methods for small RNA targets. A recent study introduced a rolling circle amplification (RCA)–assisted CRISPR/Cas9 assay for *Echinococcus multilocularis*-derived miRNA let-7-5p detection, achieving an ultralow limit of detection of 10 aM—far surpassing conventional RT-qPCR and RCA sensitivity [33]. The method integrates padlock probe ligation, RCA, and CRISPR/Cas9-mediated cleavage for fluorescence-based readout and can be performed isothermally (25–37 °C) without complex instruments, offering a low-cost (~ $2 per test), field-deployable diagnostic alternative. This system demonstrated 97–100% diagnostic sensitivity and specificity in animal plasma and can visually display results via portable fluorescence or UV observation [33]. Such hybrid nucleic-acid amplification and CRISPR-based approaches hold strong potential for adapting to schistosome miRNA detection, bridging laboratory innovation with real-world surveillance in endemic settings.

Moreover, many endemic regions also lack the infrastructure and technical workforce necessary for molecular diagnostics, resulting in a disconnect between research progress and local health implementation. Even when assays are successfully developed in advanced laboratories, the absence of supportive infrastructure, supply chains, and maintenance systems prevents their effective deployment at the community level [34]. In addition, molecular diagnostics remain largely excluded from national schistosomiasis surveillance programs, where microscopy and antigen detection still dominate, reflecting limited policy support and coordination [35]. This situation is compounded by profound global inequities: high-income countries and well-funded institutions continue to advance diagnostic innovation, whereas the populations most affected by schistosomiasis have minimal access to these technologies [36]. Without deliberate efforts to strengthen infrastructure, promote policy integration, and ensure equitable accessibility, miRNA-based diagnostics risk remaining confined to the laboratory—further widening health disparities instead of bridging them.

## Future perspective

Advancing schistosome miRNA diagnostics from conceptual promise to practical implementation demands focused innovation in technology standardization and accessibility. Future research should prioritize the development of simplified, low-cost, and field-ready diagnostic systems capable of operating independently of laboratory infrastructure [5]. Equally important is the harmonization of laboratory workflows—covering RNA extraction, reference standards, and data reporting—to ensure reproducibility and cross-study comparability. Establishing an open-access global database for *Schistosoma* miRNA signatures would further accelerate assay validation and epidemiological mapping. On the policy level, embedding molecular diagnostics into national schistosomiasis control programs will require sustained collaboration among governments, WHO, and industry stakeholders to support technology transfer, workforce training, and long-term funding mechanisms [36].

Ultimately, translating diagnostic innovation into public health impact will depend on inclusive frameworks that combine molecular science with community engagement, ensuring that new technologies are both scientifically sound and socially adoptable in the regions most affected by the disease.

## Conclusions

Research on schistosome-derived miRNAs has introduced a new molecular dimension to parasitology and highlighted their potential as diagnostic biomarkers for neglected tropical diseases. However, breakthroughs hold real value only when translated into tools accessible to those most affected. If miRNA-based technologies remain confined to advanced laboratories, their impact on disease control will be limited.

This article highlights three key contributions: (1) summarizing the biological and diagnostic potential of schistosome miRNAs; (2) identifying systemic barriers from laboratory to field; and (3) outlining future directions that integrate technology, policy, and social engagement. In our opinion, the path forward requires a shift in focus: from laboratory novelty to field applicability, from proof-of-concept to policy integration, and from scientific promise to public health equity. Only then can schistosome miRNA diagnostics evolve from experimental biomarkers into practical tools that contribute meaningfully to global schistosomiasis elimination.

## Data Availability

Not applicable.

## Abbreviations

CAA: Circulating anodic antigen
CCA: Circulating cathodic antigen
EVs: Extracellular vesicles
LAMP: Loop-mediated isothermal amplification
NGS: Next-generation sequencing
qRT-PCR: Quantitative reverse transcription PCR
RCA: Rolling circle amplification
RPA: Recombinase polymerase amplification
WHO: World Health Organization
